# Host Suitability of Lettuce and Bean Germplasm for *Meloidogyne incognita* and *M. javanica* Isolates from Spain

**DOI:** 10.3390/plants13010038

**Published:** 2023-12-21

**Authors:** Ariadna Giné, Anna Sanz-Prieto, Luiz Antonio Augusto Gomes, Alejandro Expósito, Nuria Escudero, Francisco Javier Sorribas

**Affiliations:** 1 Department of Agri-Food Engineering and Biotechnology, Barcelona School of Agri-Food and Biosystems Engineering, Baix Llobregat Campus, Universitat Politècnica de Catalunya, Esteve Terradas 8, Castelldefels, 08860 Barcelona, Spain; anna.prieto0@gmail.com (A.S.-P.); alejandro.exposito@upc.edu (A.E.); nuria.escuder@gmail.com (N.E.); 2 Department of Agronomy, School of Agriculture, Patos de Minas Campus, University Center of Patos de Minas, Rua Major Gote, 808, Patos de Minas 38700-207, MG, Brazil; laagomes.ufla@gmail.com

**Keywords:** equilibrium point, *Lactuca sativa* L., multiplication rate, *Phaseolus vulgaris* L., reproduction index, root-knot nematodes, tolerance limit

## Abstract

*Meloidogyne* spp. are an important threat to horticulture and cause substantial yield losses. Plant resistance is an alternative control method for chemical nematicides. This study highlights the host suitability of the lettuces cultivars Grand Rapids and Salinas 88 and the beans cultivars Aporé, Cornell 49242, Macarrão Atibaia and Ouro Negro to four *Meloidogyne incognita* and seven *M. javanica* isolates from Spain in a pot experiment. Moreover, the response of these cultivars to increasing *M. incognita* densities (*Pi*) was assessed in a plastic greenhouse. The lettuce cultivar Regina 71 and the bean cultivar Bolinha were included as susceptible standards for comparison. It was found that Grand Rapids and Salinas 88 lettuces were resistant to the most nematode isolates in the pot experiment but were classified as slightly and moderately resistant, respectively, in the plastic greenhouse at increasing *Pi.* Regarding the beans, Aporé was resistant to the majority of the *Meloidogyne* isolates whereas Macarrão Atibaia and Ouro Negro were slightly resistant and Cornell 49242 was susceptible in the pot experiment. In the plastic greenhouse, Aporé was the only cultivar able to effectively suppress the nematode reproduction irrespective of *Pi*, while Ouro Negro became less resistant as *Pi* increased. These results play an important role in enhancing the effective and ecofriendly *Meloidogyne* management strategies.

## 1. Introduction

One of the world’s leading areas horticultural production is the Mediterranean basin, where high-value fruiting vegetable crops such as Solanaceae (tomato, pepper and eggplant) and Cucurbitaceae (cucumber, melon and zucchini) are grown [1]. Other crops that are commonly included in rotation sequences with these belong to the Compositae and Fabaceae families, mainly lettuce and beans, with Spain being the largest and second largest EU producer, respectively [2]. Meanwhile, one of the main soil-borne pathogens that limit vegetable production worldwide consists of the root-knot nematodes (RKNs), *Meloidogyne* spp. Within the genus, the tropical *Meloidogyne incognita*, *M. javanica* and *M. arenaria* are the most common RKN species and can cause crop yield losses [3,4,5]. To control RKNs, farmers have relied heavily on chemical fumigants and nematicides, but their use has been restricted due to human and animal toxicity and environmental contamination. Current legislation seeks to reduce the dependence on chemical pesticides to 50% by 2030 and to promote the use of non-chemical alternatives via Directive 2009/12/128/EC and by the EU Farm to Fork Strategy.

Plant resistance is a sustainable method of control that improves crop yield [6], is compatible with other control methods, reduces the nematode population growth rate and, consequently, reduces the crop losses of the following crop at the rotation cycle [3]. However, some limitations concerning the use of plant resistance must be taken into account. Resistant cultivars and/or rootstocks are only commercially available for a few horticultural crops, such as tomato, pepper, watermelon and eggplant. Moreover, some resistance genes, like the Mi 1.2 gene in tomato, fail at constant soil temperatures above 28 °C [7] but not when the high soil temperature peaks during the day because the expression is recovered with time as soil temperature decreases [8]. Also, the repeated cultivation of resistant tomato cultivars carrying the Mi 1.2 gene may allow for the development of virulent RKN populations or species [9,10,11]. The effectiveness and durability of a given resistance source can be maximized if it is included in rotation sequences with plants carrying different resistance genes and/or with non- or poor hosts [12]. The host status of a given plant to a given nematode species is defined by the relationship between the nematode densities at the end of the crop or experiment (*Pf*) and the nematode densities at the beginning of the crop or experiment (*Pi*) [13,14]. *Pi*, in the absence of limiting factors, will increase and the multiplication rate (*Pf*/*Pi*) will be at a maximum at low *Pi* values. As *Pi* increases, there is competition, a scarcity of food and a decrease in the multiplication rate, which tends to stabilize around the equilibrium density (*E*: *Pf* = *Pi*), at which point the plant can supply enough food to maintain the population density at planting.

Characterization of the level of plant germplasm resistance to RKN populations or isolates provides a broad view of the possible response in field conditions since the result of the plant–nematode interaction is dependent on the genetic background. Some studies have found that certain lettuce cultivars are resistant to *M. incognita* and *M. javanica* [15,16,17,18,19,20,21,22] populations and/or races, as well as bean cultivars [23,24,25,26,27]. So, in this study, some experiments were conducted to determine the level of resistance of three lettuce and five bean cultivars against eleven RKN isolates from Spain in pot experiments, and against an *M. incognita* isolate in a plastic greenhouse.

## 2. Results

### 2.1. Screening Lettuce and Bean Cultivars for Resistance against Meloidogyne Isolates in Pot Experiments

In general, *Meloidogyne* spp. produced fewer (*p* < 0.05) eggs on the lettuce cultivars Salinas 88 and Grand Rapids than on Regina 71. Both former lettuce cultivars were resistant to all the tested isolates of RKNs after one nematode generation in the pot test. The number of eggs per plant on these cultivars was 2.7 and 6.8% of the eggs observed on the susceptible Regina 71 cultivar, respectively (Table 1). The nematode reproduction on the lettuce cultivars did not differ (*p* > 0.05) between *M. incognita* and *M. javanica* when the nematode isolates were grouped by RKN species.

The response of the lettuce cultivars varied according to the nematode isolate (Table 2). Grand Rapids and Salinas 88 were resistant to 9 and 10 of the 11 RKN isolates assessed, respectively. Both these cultivars were susceptible to the Curas isolate, which, in contrast, reproduced poorly on the susceptible cultivar Regina 71. Also, the cultivar Grand Rapids responded as susceptible to the Al05 isolate.

In general, the Grand Rapids cultivar was less resistant than Salinas 88 according to the resistance levels observed; that is, Grand Rapids responded as resistant, moderately resistant or slightly resistant against 36.3%, 36.3% and 9% of the RKN isolates, respectively, whereas Salinas 88 responded as highly resistant, resistant or moderately resistant against 27.2%, 36.3% and 27.2% of the RKN isolates, respectively.

Regarding beans, the cultivar Aporé was resistant to all isolates after one nematode generation in the pot test. The cultivars Macarrão Atibaia and Ouro Negro were characterized as slightly resistant to all RKN isolates, hosting, respectively, 33.4% and 47.5% of the eggs per plant found on the susceptible cultivar Bolinha. The Cornell cultivar was classed as susceptible to the RKN isolates, as it hosted 81% of the eggs produced per plant on the susceptible cultivar Bolinha (Table 3). The nematode reproduction on bean cultivars did not differ (*p* > 0.05) between *M. incognita* and *M. javanica* when the nematode isolates were grouped by RKN species.

The responses of the bean cultivars differed according to the nematode isolates. Aporé responded as highly resistant or resistant to 7 of the 11 RKN isolates. Macarrão Atibaia and Ouro Negro had similar responses to five of the isolates: susceptible to MJ05, MJAl101 and Viator; slightly resistant to Adra and resistant to Curas isolates. Against MiAl09, Ouro Negro responded as moderately resistant, whereas Macarrão Atibaia was susceptible. Macarrão Atibaia responded as moderately resistant to Al05, Amat, MiAl30, P Almeria and P Murica whereas Ouro Negro responded as resistant to Amat, slightly resistant to Al05 and P Almeria and susceptible to MiAl 30 and P Murica isolates. Cornell was susceptible to 81.8% of the isolates, slightly resistant to P Murcia and resistant to Curas (Table 4).

### 2.2. Screening Lettuce and Bean Cultivars for Resistance against M. incognita in Plastic Greenhouse

In the plots cropped with lettuce, *Pi* ranged from 218 to 7613 J2 per 500 cm³ of soil. At the highest *Pi* level, plants of the most susceptible cultivar, Regina 71, died. These plants were not included for the statistical analysis of the galling index and eggs per plant. Despite this, the gall index and the eggs per plant in the Grand Rapids and Salinas 88 cultivars were significantly lower (*p* < 0.05) than those in the surviving Regina 71 plants. The nematode produced fewer (*p* < 0.05) eggs on Salinas 88 than in Grand Rapids, which were classified as moderately and slightly resistant, respectively (Table 5).

**Figure 1:**
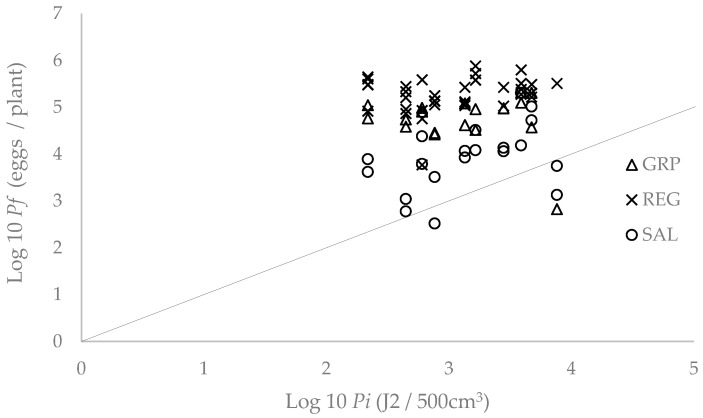
Relationship between the initial (*Pi*) and final (*Pf*) population densities of *M. incognita* in the resistant lettuce cultivars Grand Rapids (GRP) and Salinas 88 (SAL) and the susceptible cultivar Regina 71 (REG) 49 days after transplanting in the plastic greenhouse.

The maximum multiplication rate of *M. incognita* in all the lettuce cultivars occurred at the lowest *Pi* (218 J2 per 500 cm^−3^ of soil). The maximum multiplication on the Salinas 88 and Grand Rapids cultivars was, respectively, 1.9% and 27.6% that achieved in the susceptible cultivar Regina 71. The equilibrium densities of the nematode isolate from the cultivars Salinas 88 and Grand Rapids were, respectively, 21.2% and 32.4% that from the susceptible Regina 71. The maximum multiplication rate in Grand Rapids was higher than in Salinas 88 but the maximum nematode density and equilibrium density were similar (Table 6; Figure 1).

Concerning the bean crop, *Pi* ranged from 181 to 5749 J2 per 500 cm³ of soil. The bean cultivars Aporé and Ouro Negro showed lower gall indexes and had a lower root weight and fewer eggs per plant than the susceptible cultivar Bolinha. Aporé was resistant (RI = 7.4%) and Ouro Negro was slightly resistant (RI = 35.3%). The cultivar Macarrão Atibaia did not differ (*p >* 0.05) from Bolinha in any of the parameters measured, being susceptible to the *M. incognita* isolate Agròpolis (RI = 81.8%) (Table 7).

**Figure 2:**
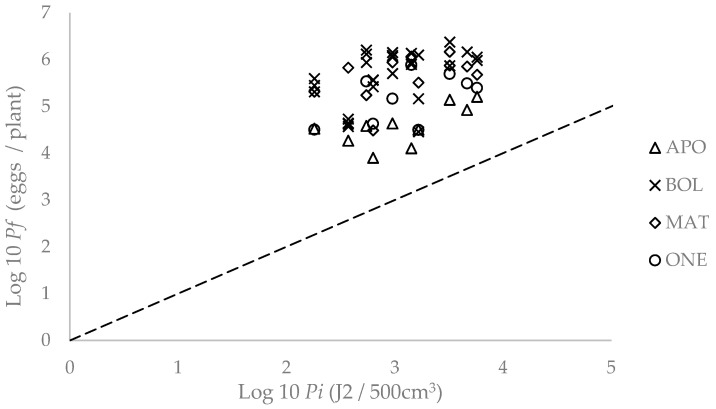
Relationship between the initial (*Pi*) and final (*Pf*) population densities of *M. incognita* in the bean cultivars Aporé (APO), Macarrão Atibaia (MAT) and Ouro Negro (ONO), and in the susceptible bean cultivar Bolinha (BOL) 54 days after sowing in plastic greenhouse.

The maximum multiplication rate of the nematode occurred at the lowest *Pi* (181 J2 per 500 cm^−3^ of soil) in all bean cultivars. In the cultivars Aporé, Macarrão Atibaia and Ouro Negro, *a* was 11.4%, 70.3% and 10.8% of that registered in the susceptible cultivar Bolinha, respectively. The equilibrium density of the nematode in the cultivars Aporé, Macarrão Atibaia and Ouro Negro was 6.7%, 62% and 32.5% of that in the susceptible cultivar Bolinha, respectively (Table 8; Figure 2).

## 3. Discussion

The results from the present study confirmed the resistance of the lettuce cultivars Grand Rapids and Salinas 88 to the majority of the RKN isolates from Spain assessed. However, Salinas 88 demonstrated a greater resistance level than Grand Rapids against the *Meloidogyne* isolates. While Salinas 88 performed as highly resistant and resistant to six nematode isolates, Grand Rapids was only resistant to four of them. These lettuce cultivars were previously reported as resistant to *Meloidogyne incognita* and *M. javanica* from Brazil [15,17,18,20,21,22]. Moreover, the Grand Rapids cultivar was reported as resistant to *Meloidogyne enterolobii* [29].

In our study, greater differences in reproduction between RKN isolates on the susceptible cultivar Regina 71 were observed than on the resistant cultivars Grand Rapids or Salinas 88, indicating that both the genetic background of the RKN isolate and that of the cultivar can play an important role in the plant–nematode interaction [30]. For example, the RKN isolate Curas reproduced poorly on all lettuce cultivars, including the susceptible cultivar Regina 71. Several studies have reported variability in the reproductive ability of RKN populations or isolates in a given plant species [30,31,32,33]. In addition, the isolate Al05 was virulent to Grand Rapids, probably due to the genetic nature of the resistance in this cultivar. Maluf et al. [17] reported that Grand Rapids resistance is predominantly due to additive genes and has incomplete penetrance and variable expressivity. This allele was named *Me* by Gomes et al., 2000 [15]. In comparison to Grand Rapids, the Salinas 88 resistance gene has partial dominance and seems to have an n-allelism of the major genes controlling resistance, and it has been suggested to name this allelism that controls resistance to *M. incognita* as *Me2* [20].

In the plastic greenhouse conditions in which the lettuce cultivars were subjected to an increasing nematode density at transplanting, Salinas 88 responded as resistant at low *Pi*, according to the maximum multiplication rate, but moderately resistant (RI = 10.5%) considering the whole range of *Pi* assessed. Consequently, for the optimal use of this lettuce cultivar under field conditions, it is important to consider nematode densities at planting, which are optimal after cropping non-host, poor-host or resistant cultivars, leading to a low nematode density before planting the lettuce.

The bean screening experiment showed that cultivar Aporé was resistant (RI = 7.4%) to most RKN isolates from Spain. Previous studies have reported its resistance [25,26,27,29]. However, the cultivars Ouro Negro, Macarrão Atibaia and Cornell responded as susceptible to 45.4%, 36.4% and 90.9% of the nematode isolates, respectively. This confirms the variable response of the cultivar according to the plant germplasm–RKN isolate combination, as demonstrated by Ferreira et al. [26], Oliveira et al. [27] and Chen and Roberts [34]. Under plastic greenhouse conditions, with a gradient of *Pi*, the cultivar Aporé maintained its level of resistance (RI = 7.4%); Ouro Negro responded as slightly resistant (RI = 35.3%); and Macarrão Atibaia was susceptible (RI = 81.8%).

The RKN resistance in beans has been attributed to different resistance genes depending on the plant germplasm studied. Indeed, in cultivar Aporé, the resistance to *M. incognita* and *M. javanica* was under control of a single locus with incomplete dominance [26], but it was conferred by the single dominant gene *Me1* in bean lines A315 and A445, by unidentified recessive genes in lines Alabama no. 1 and PI 165435 or by one dominant gene and one recessive gene (*Me2me3*) in line PI 165426 [35]. Chen and Roberts [34] also proposed a single dominant gene in cultivar NemaSnap to *M. hapla*.

The results of this study provide information on the resistance of lettuce and bean germplasm to *M. incognita* and *M. javanica*, which therefore could be included in rotation sequences for the nematode management as an alternative control method for chemical nematicides. According to the results of this study, the lettuce cultivar Salinas 88 and the bean cultivar Aporé would be recommended, contributing to reducing the increase in RKN population densities and consequently the cost of control. Cropping these cultivars after non-host, poor-host or resistant cultivars that lead to low *Pi* will suppress the raising of the nematode population in comparison with a susceptible cultivar, and this effect can be maximized depending on the date of transplanting. Indeed, in northeastern Spain, lettuce can act as trap crop when it is transplanted in the middle of October or November because the nematode can infect roots but does not reproduce at the end of the crop, reducing nematode densities in the soil between 20 and 50% [36]. However, this control method needs the support of the nematode phenology models to have information of the accumulated degree-days to foresee the end of the crop when the plant has to be uprooted or destroyed. Concerning the bean crop, it can be conducted all over the year while soil temperatures are between 15 °C and 30 °C. Then, the use of phenology models could be useful for cropping bean in periods in which the nematode can penetrate roots but not achieve reproduction or in which the number of generations that the nematode can complete is reduced [37].

In short, this study contributes to provide information on lettuce and bean germplasm able to reduce *M. incognita* and *M. javanica* to be included in a crop rotation sequence as an alternative to chemical control. Moreover, the inclusion of different resistance genes in crop rotation sequences can contribute to the durability of each single resistance gene despite having been previously selected for their virulence to a specific resistance gene [12,38]. Furthermore, if selection for virulence of a given specific resistance gene did occur, it would not compromise other resistance genes [39].

## 4. Materials and Methods

### 4.1. Plant Material

Three lettuce cultivars (Grand Rapids, Regina 71 and Salinas 88) and five bean cultivars (Aporé, Bolinha, Cornell 49242, Macarrão Atibaia and Ouro Negro) were used in this study. The lettuce cultivar Regina 71 and the bean cultivar Bolinha were included as susceptible control standard for comparison. The characteristics of the lettuce and bean cultivars are shown in Table 9 and Table 10, respectively.

### 4.2. Nematode Isolates

Twelve *Meloidogyne* isolates from three vegetable-growing areas of Spain were reared in the susceptible tomato (*Solanum lycopersicum*) cultivar Durinta (Seminis Seeds, St. Louis, MO, USA) from a single egg mass. Afterward, the tomato cultivar Durinta was inoculated to produce enough inoculum to carry out the pot experiments. The *Meloidogyne* species were identified by perineal patterns and molecular SCAR–PCR markers [40].

The nematode inoculum used in pot experiments consisted of second-stage juveniles (J2) that emerged from eggs previously extracted from tomato roots using the Hussey and Barker’s method [41] by blender maceration in 5% of commercial bleach solution (35 g L^−1^ NaOCl) for 10 min. The egg suspension was passed through a 75 µm pore sieve to remove organic material and passed through a 25 µm pore sieve to retain the egg suspension. The egg suspension was then placed in a Baermann tray [42] for two weeks at room temperature. Any J2 recovered in the first 24 h were discarded but, later, J2 were collected periodically, counted and maintained at 9 °C until inoculation. The nematode inoculum was added via two holes at 1 cm from the stem and at a depth of 3 cm.

The soil in the plastic greenhouse was infested with the Agròpolis isolate cropping the susceptible tomato cultivar Durinta inoculated with the nematode for three years before the experiment. The *Meloidogyne* species used in the pot and greenhouse experiments and their origin are shown in Table 11.

### 4.3. Screening Lettuce and Bean Cultivars for Resistance against Meloidogyne Isolates in Pot Experiments

Lettuce seeds were sown in trays containing vermiculite and then maintained at 25 °C with 16:8 light:darkness cycle in a growth chamber until the second leaf appeared. The plantlets were transplanted individually to seedling trays containing vermiculite and fertilized with Hoagland solution. Three weeks later, each plant was transplanted in a 0.5 L pot containing sterile river sand. For the beans, two seeds were sown in 1 L pot containing sterile river sand and placed on benches in a greenhouse. One week later, one plant per pot was left.

Two weeks after sowing the beans and one week after lettuce transplantation, the pots were inoculated, respectively, with 1000 or 500 s stage juvenile (J2) per pot. The nematode inoculum was placed in two holes, 2 cm apart, on opposite sides of the steam at 3 cm depth. The plants were distributed at random, watered by drip irrigation as needed and fertilized with a slow-release fertilizer (Osmocote plus ©, Atlanta, GA, USA). Each combination cultivar–nematode isolate was replicated 10 times.

The soil temperature and the water content were recorded with 5 TE sensors (Decagon devices, Pullman, WA, USA) at 1 h intervals at 8 cm depth from the pots along the benches. The experiments were carried out from 11 May to 27 June (48 days) with a final temperature accumulation of 1281 °C for lettuce (base temperature, *Tb* = 0 °C) and from 16 May to 7 July (53 days) with a final accumulation of 1459 °C for bean.

At the end of the experiments, the roots were washed in tap water, gently dried and weighed. The number of eggs per plant was determined by the Hussey and Barker method [41], extracting the eggs from the complete root system by blender maceration in a 10% bleach solution (35 g L^−1^ NaOCl) for 10 min. The reproduction index (RI) was calculated as the percentage of the number of eggs per plant produced on a given cultivar divided by those produced on the susceptible Regina 71 cultivar for lettuce or the susceptible Bolinha cultivar for beans. The level of resistance of each cultivar was classified as highly resistant (RI < 1%), resistant (1% ≤ RI ≤ 10%), moderately resistant (10% < RI ≤ 25%), slightly resistant (25% < RI ≤ 50%) or susceptible (RI > 50%) in accordance with the categorization of Hadisoeganda and Sasser [28].

### 4.4. Screening Lettuce and Bean Cultivars for Resistance against M. incognita in Plastic Greenhouse

The three cultivars of lettuce and four cultivars of bean (Cornell 49242 was not included) were used in a plastic greenhouse experiment infested with the isolate Agròpolis of *M. incognita*.

The soil was sandy loam with 83.8% sand, 6.7% silt and 9.5% clay; pH 8.7; 1.8% organic matter; and 0.5 dS/m of electric conductivity. Plots 0.5 m wide and 2.5 m long were sampled to determine the initial population density (*Pi*). Composite soil samples consisted of 4 cores taken with an auger (2.5 cm diameter) from the first 30 cm of soil. The soil was homogenized and J2 were extracted from 500 cm³ of soil using Baermann trays [42]. After one week, the J2 suspension was passed through a 25 µm sieve and counted. In each plot, all cultivars were planted but the resistant cultivars were arranged between the standard susceptible ones. In total, ten plots with different *Pi* were cultivated.

Lettuce was sown, as previously described, and transplanted on 13 May. The same day, bean seeds were sown directly in the greenhouse. The lettuce crop lasted 49 days and the bean crop 54 days. Plants were watered as needed via drip system and fertilized weekly with a solution consisting of NPK (15-5-30) at 31 kg ha^−1^ and iron chelate and micronutrients at 0.9 kg ha^−1^.

The soil temperatures and water content at 15 cm depth were recorded daily at 30 min intervals with 5 TM probes (Decagon Devices Inc., Pullman, WA, USA).

The experiment finished after 1168 °C and 1299 °C DD (*Tb* = 0 °C) for lettuce and beans, respectively. The gall index was assessed on a 0 to 10 scale, where 0 means healthy root system with no galls and 10 means dead root system and plant [43]. After that, the roots were chopped into 2 cm long fragments, and eggs were extracted as in the previous experiment. Also, the RI was calculated and the level of resistance was categorized. In addition, the maximum multiplication rate (*a*) was estimated by the slope of the linear regression between *Pf* and the lowest values of *Pi* according to *Pf* = *aPi* [13]. The maximum nematode density (*M*) was estimated from the experimental data, and the equilibrium density (*E*) was calculated according to *M* = *aE*/(*a* − 1) [44].

### 4.5. Statistical Analysis

Statistical analysis was performed using R statistical software version 4.3.1 (R Foundation for Statistical Computing, Vienna, Austria). Data for the root weight, gall index and eggs per plant were analyzed by the non-parametric Kruskal–Wallis test because they did not fit a normal distribution. All the data from each crop were compared between cultivars for each nematode isolate to establish the host suitability and between nematode isolates per cultivar to know the putative variability due to the genetic background of the nematode isolate. When the non-parametric analysis was significant (*p* < 0.05), groups were separated by the Dunn test (*p* < 0.05). Finally, nematode isolates were grouped per RKN species and compared between them per crop cultivar to determine their parasitic capacity using the non-parametric Wilcoxon test.

## 5. Conclusions

This study confirms the resistance of the lettuce cultivars Grand Rapids and Salinas 88 to *M. incognita* and *M. javanica* isolates from Spain as demonstrated in all the experiments. However, the resistance of Grand Rapids was influenced by *Pi*. The bean cultivar Aporé exhibited resistance to most of the *M. incognita* and *M. javanica* isolates assessed, and was the only bean cultivar that effectively suppressed nematode population growth regardless of *Pi*. The other bean cultivars were less resistant in pot experiments and were susceptible when cultivated at plastic greenhouse or were influenced by *Pi* as in the case of Ouro Negro.

## Figures and Tables

**Table 1:** Fresh root weight, number of eggs per plant, resistance index (RI) and resistance level (RL) of the resistant lettuce cultivars Grand Rapids and Salinas 88 and of the susceptible cultivar Regina 71 against 11 *Meloidogyne* isolates after 48 days of inoculation.

Cultivar	Fresh Root Weight (g)	Number of Eggs Plant^−1^	RI (%) ^a^	RL ^b^
Grand Rapids	5.32 ± 0.21 B	306 ± 50 B	6.8%	R
Salinas 88	5.67 ± 0.38 B	121 ± 21 B	2.7%	R
Regina 71	7.64 ± 0.56 A	4490 ± 1061 A		

Data are presented as mean ± standard error of 110 replicates. Data in the same column followed by different letters are significantly different (*p* < 0.05) according to the Dunn test. ^a^ Resistance index: number of eggs on the tested cultivar as a percentage of those on the susceptible. ^b^ Resistance level: HR = highly resistant (RI < 1%), R = resistant (1% ≤ RI ≤ 10%), MR = moderately resistant (10%< RI ≤ 25%), SR = slightly resistant (25% < RI ≤ 50%) or S = susceptible (RI > 50%) in accordance with the categorization of Hadisoeganda and Sasser (1982) [28].

**Table 2:** Number of eggs per plant, resistance index (RI) and resistance level (RL) for lettuce cultivars Grand Rapids, Salinas 88 and the susceptible cultivar Regina 71 against 11 *Meloidogyne* isolates 48 days after inoculation with 500 J2 per plant.

*Meloidogyne* Isolate	*Meloidogyne* Species	Eggs Per Plant			RI ^a^ (%) and RL ^b^	
		Regina 71	Grand Rapids	Salinas 88	Grand Rapids	Salinas 88
Adra	*M. javanica*	2517 ± 926 A bc	89 ± 25 C a	302 ± 124 B a	3.5 R	12.0 MR
Al05	*M. javanica*	1484 ± 680 A bc	785 ± 259 A a	103 ± 31 A b	52.9 S	7.0 R
Amat	*M. javanica*	30,862 ± 8613 A a	573 ± 274 B a	73 ± 37 C b	1.9 R	0.23 HR
Curas	*M. javanica*	118 ± 86 A c	199 ± 53 A a	178 ± 36 A ab	168.8 S	151.1 S
MJ05	*M. javanica*	3230 ± 1095 A bc	527 ± 234 B a	133 ± 119 C ab	16.3 MR	4.1 R
MJAl101	*M. javanica*	5554 ± 2682 A bc	145 ± 87 B a	32 ± 17 B b	2.6 R	0.6 HR
MiAl09	*M. incognita*	412 ± 267 A c	135 ± 67 A a	46 ± 18 A b	32.7 SR	11.1 MR
MiAl30	*M. incognita*	658 ± 388 A c	99 ± 44 A a	39 ± 32 B b	15.0 MR	6.0 R
P Almeria	*M. incognita*	1006 ± 821 A c	154 ± 67 A a	159 ± 90 A ab	15.3 MR	18.3 MR
P Murcia	*M. incognita*	4035 ± 2041 A bc	564 ± 453 A a	193 ± 40 A ab	14.0 MR	4.8 R
Viator	*M. javanica*	8069 ± 3820 A b	322 ± 125 B a	56 ± 24 C b	4.0 R	0.70 HR

Data are the mean ± standard error of 10 replicates of each combination cultivar–RKN isolate. Data in the same row for eggs per plant followed by different uppercase letters are significantly different (*p* < 0.05) according to the Dunn test, indicating differences between lettuce cultivars per nematode isolate. Data in the same column followed by different lowercase letters are significantly different (*p* < 0.05) according to the Dunn test, indicating differences between nematode isolates for a given lettuce cultivar. ^a^ Resistance index: number of eggs on the tested cultivar as a percentage of those on the susceptible; ^b^ Resistance level: HR= highly resistant (RI < 1%), R = resistant (1% ≤ RI ≤ 10%), MR = moderately resistant (10% < RI ≤ 25%), SR = slightly resistant (25% < RI ≤ 50%) or S = susceptible (RI > 50%) in accordance with the categorization of Hadisoeganda and Sasser (1982) [28].

**Table 3:** Fresh root weight, number of eggs per plant, resistance index (RI) and resistance level (RL) of the bean cultivars Aporé, Cornell Macarrão Atibaia and Ouro Negro and the susceptible cultivar Bolinha against all 11 *Meloidogyne* isolates 53 days after inoculation with 1000 J2 per plant.

Cultivar	Fresh Root Weight (g)	Number of Eggs Plant^−1^	RI (%) ^a^	RL ^b^
Apore	8.00 ± 0.38 B	1090 ± 338 C	7.4%	R
Bolinha	9.56 ± 0.38 A	14,650 ± 2162 A		
Cornell	10.42 ± 0.5 A	11,871 ± 2084 A	81.0%	S
Macarrão Atibaia	7.26 ± 0.53 B	4894 ± 940 BC	33.4%	SR
Ouro negro	7.92 ± 0.43 B	6961 ± 1306 B	47.5%	SR

Data are presented as mean ± standard error of 110 replicates. Data in the same column followed by different letters are significantly different (*p* < 0.05) according to the Dunn test. ^a^ Resistance index: number of eggs on the tested cultivar as a percentage of those on the susceptible. ^b^ Resistance level: HR= highly resistant (RI < 1%), R = resistant (1% ≤ RI ≤ 10%), MR = moderately resistant (10%< RI ≤ 25%), SR = slightly resistant (25% < RI ≤ 50%) or S = susceptible (RI > 50%) in accordance with the categorization of Hadisoeganda and Sasser (1982) [28].

**Table 4:** The number of eggs per plant, resistance index (RI) and resistance level (RL) of bean cultivars. Aporé, Cornell Macarrão Atibaia and Ouro Negro; the susceptible cultivar Bolinha against 11 *Meloidogyne* isolates 53 days after inoculation with 1000 J2 per plant.

*Meloidogyne* Isolate	*Meloidogyne* Species	Eggs Per Plant					RI ^a^ (%) and RL ^b^			
		Bolinha	Aporé	Cornell	Macarrão Atibaia	Ouro Negro	Aporé	Cornell	Macarrão Atibaia	Ouro Negro
Adra	*M. javanica*	7458 ± 2045 A cd	5667 ± 2717 A a	11,560 ± 5161 A bc	3542 ± 1595 A bc	3437 ± 1159 A bcd	76.0 S	155.0 S	47.5 SR	46.1 SR
Al05	*M. javanica*	10,673 ± 2839 A cd	54 ± 24 C b	5688 ± 2110 AB c	1725 ± 480 BC c	4262 ± 2297 BC bcd	0.5 HR	53.3 S	16.2 MR	39.9 SR
Amat	*M. javanica*	9921 ± 2694 B cd	1367 ± 469 B b	30,096 ± 10,623 A a	1131± 498 B c	342 ± 209 B d	13.8 MR	303.3 S	11.4 MR	3.5 R
Curas	*M. javanica*	30,792 ± 12,717 A ab	162 ± 58 B b	2728 ± 867 B c	587 ± 277 B c	2375 ± 420 B cd	0.5 HR	8.6 R	1.9 R	7.7 R
MJ05	*M. javanica*	9530 ± 4323 AB cd	65 ± 47 B b	16,066 ± 3559 A abc	7322 ± 3836 AB bc	17,034 ± 5835 A a	0.7 HR	168.6 S	76.8 S	178.7 S
MJAl101	*M. javanica*	4319 ± 1234 AB cd	131 ± 46 B b	2687 ± 1343 AB c	5663 ± 3887 AB bc	11,009 ± 5113 A abcd	3.0 R	62.2 S	131.1 S	254.9 S
MiAl09	*M. incognita*	2227 ± 379 AB cd	85 ± 35 B b	6654 ± 3413 A c	6840 ± 2746 A bc	515 ± 285 B d	3.8 R	299.0 S	307.1 S	23.1 MR
MiAl30	*M. incognita*	15,015 ± 2618 A bcd	56 ± 27 C b	5688 ± 2110 AB c	3099 ± 1041 BC bc	9229 ± 2949 AB abcd	0.5 HR	53.3 S	20.6 MR	61.5 S
P Almeria	*M. incognita*	45,628 ± 9521 A a	5166 ± 2273 C a	29,378 ± 13,965 AB ab	9915 ± 2663 BC b	14,109 ± 3725 BC ab	11.3 MR	64.0 S	21.7 MR	30.9 SR
P Murcia	*M. incognita*	366 ± 158 AB d	149 ± 70 AB b	126 ± 49 BC c	52 ± 23 C c	388 ± 81 A d	41.0 SR	34.5 SR	14.2 MR	106.0 S
Viator	*M. javanica*	17,825 ± 4757 A bc	21 ± 12 B b	17,458 ± 7204 AB abc	20,558 ± 8957 A a	14,026 ± 10,296 AB abc	0.1 HR	97.9 S	115.3 S	78.7 S

Data are presented as the mean ± standard error of 10 replicates of each combination cultivar in the RKN isolate. Data in the same row for eggs per plant followed by different uppercase letters are significantly different (*p* < 0.05) according to the Dunn test, indicating differences between lettuce cultivars per nematode isolate. Data in the same column followed by different lowercase letters are significantly different (*p* < 0.05) according to the Dunn test, indicating differences between nematode isolate for a given lettuce cultivar. ^a^ Resistance index: number of eggs on the tested cultivar as a percentage of those on the susceptible; ^b^ Resistance level: HR = highly resistant (RI < 1%), R = resistant (1% ≤ RI ≤ 10%), MR = moderately resistant (10% < RI ≤ 25%), SR = slightly resistant (25% < RI ≤ 50%) or S = susceptible (RI > 50%) in accordance with the categorization of Hadisoeganda and Sasser (1982) [28].

**Table 5:** Gall index, fresh root weight, number of eggs per plant, resistance index (RI) and resistance level (RL) of the resistant lettuce cultivars Grand Rapids and Salinas 88 and the susceptible cultivar Regina 71 after 49 days of development in *M. incognita*-infested soil.

Cultivar	Galling Index	Root Weight	Eggs Plant^−1^ (×10^3^)	RI (%) ^a^	RL ^b^
Grand Rapids	2.8 ± 0.2 B	9.6 ± 0.6 B	81.4 ± 12.9 B	33.1	SR
Salinas 88	2.8 ± 0.2 B	9.7 ± 0.9 B	25.9 ± 10.7 C	10.5	MR
Regina 71	5.3 ± 0.3 A	23.8 ± 1.4 A	245.7 ± 27.6 A		

Data are the mean ± standard error of 40 replicates for Regina 71, 20 replicates for Grand Rapids and 20 replicates for Salinas 88. Data in the same column followed by different letters are significantly different (*p* < 0.05) according to the Dunn test. ^a^ Resistance index: number of eggs on the tested cultivar as a percentage of those on the susceptible. ^b^ Resistance level: HR= highly resistant (RI < 1%), R = resistant (1% ≤ RI ≤ 10%), MR = moderately resistant (10% < RI ≤ 25%), SR = slightly resistant (25% < RI ≤ 50%) or S = susceptible (RI > 50%) in accordance with the categorization of Hadisoeganda and Sasser (1982) [28].

**Table 6:** Maximum multiplication rate (*a*), maximum population density (*M*) and equilibrium density (*E*) of *Meloidogyne incognita* in lettuce cultivars Grand Rapids and Salinas 88 and the susceptible cultivar Regina 71, 49 days after transplanting on the plastic greenhouse.

Cultivar	*a*	*E* (Eggs Per Plant)	*M* (Eggs Per Plant)
Regina 71	305,812	511,033	511,035
Grand Rapids	84,430	165,578	165,580
Salinas 88	5988	108,802	108,820

**Table 7:** Gall index, fresh root weight, number of eggs per gram of root, resistance index (RI) and resistance level (RL) of the bean cultivars Aporé, Macarrão Atibaia and Ouro Negro, and the susceptible cultivar Bolinha, 54 days after sowing in a plastic greenhouse infested by *M. incognita*.

Cultivar	Galling Index	Root Weight	Eggs (×10^3^) Plant^−1^	RI (%) ^a^	RL ^b^
Aporé	1.1 ± 0.31 C	7.3 ± 0.75 B	57 ± 17 C	7.4	R
Bolinha	4.68 ± 0.41 A	16.73 ± 1.05 A	761 ± 115 A		
Macarrão Atibaia	3.36 ±0.62 AB	17.63 ± 1.30 A	623 ± 133 AB	81.8	S
Ouro Negro	2.78 ± 0.81 BC	9.46 ± 0.91 B	269 ± 827 BC	35.3	SR

Data are presented as mean ± standard error of 10 replicates for Aporé, Macarrão Atibaia and Ouro Nego and 30 replicates for Bolinha. Data in the same column followed by different letters are significantly different (*p* < 0.05) according to the Dunn test. ^a^ Resistance index: number of eggs on the tested cultivar as a percentage of those on the susceptible. ^b^ Resistance level: HR = highly resistant (RI < 1%), R = resistant (1% ≤ RI ≤ 10%), MR = moderately resistant (10% < RI ≤ 25%), SR = slightly resistant (25% < RI ≤ 50%) or S = susceptible (RI > 50%) in accordance with the categorization of Hadisoeganda and Sasser (1982) [28].

**Table 8:** Maximum multiplication rate (*a*), maximum population density (*M*) and equilibrium density (*E*) of *Meloidogyne incognita* in the bean cultivars Aporé, Macarrão Atibaia and Ouro Negro, and in the susceptible cultivar Bolinha 54 days after sowing in plastic greenhouse.

Cultivar	*a*	*E* (Eggs Per Plant)	*M* (Eggs Per Plant)
Aporé	33,500	160,995	161,000
Bolinha	293,650	2,377,162	2,377,170
Macarrão Atibaia	206,500	1,473,913	1,473,920
Ouro Negro	32,000	773,176	773,200

**Table 9:** Lettuce cultivar name, origin and *Meloidogyne* resistance in the pot and plastic greenhouse experiments.

Lettuce Cultivar	Origin	Resistance Evaluated Previously	References
Grand Rapids	USDA ^a^—improved in Brazil	*M. incognita* *M. javanica* *M. enterolobii*	[15,16,17,20,29]
Regina 71	Brazil	Susceptible cultivar (control)	[15,17]
Salinas 88	USDA ^a^—improved in Brazil	*M. incognita* *M. javanica* *M. enterolobii*	[18,20,29]

^a^ USDA U.S. Department of Agriculture.

**Table 10:** Bean cultivar name, origin and *Meloidogyne* resistance in the pot and plastic greenhouse experiments.

Bean Cultivar	Origin	Resistance Evaluated Previously	References
Aporé	Brazil	*M. enterolobii* *M. incognita* *M. javanica*	[25,26,27,29]
Bolinha	Brazil	Susceptible cultivar (control)	[26]
Cornell 49242	USDA ^a^—used in genetic improvement by SERIDA ^b^	No previous works with nematodes	
Macarrão Atibaia	Brazil	*M. enterolobii* *M. incognita* *M. javanica*	[25,26,29]
Ouro Negro	Brazil	*M. enterolobii* *M. incognita* *M. javanica*	[25,26,29]

^a^ USDA U.S. Department of Agriculture. ^b^ SERIDA. Servicio Regional de Investigación y Desarrollo Agroalimentario. Asturias (Spain).

**Table 11:** Experiment, nematode isolate code, nematode population origin and *Meloidogyne* isolate species.

Experiment	*Meloidogyne* Isolate Code	Origin	*Meloidogyne* spp.
Pot	Amat	Barcelona	*M. javanica*
	MJ 05	Barcelona	*M. javanica*
	MJ Al 101	Almería	*M. javanica*
	Al 05	Almería	*M. javanica*
	Viator	Almería	*M. javanica*
	Adra	Almería	*M. javanica*
	Curas	Murcia	*M. javanica*
	Mi Al 30	Almería	*M. incognita*
	Mi Al 09	Almería	*M. incognita*
	P Almería	Almería	*M. incognita*
	P Murcia	Murcia	*M. incognita*
Plastic greenhouse	Agròpolis	Barcelona	*M. incognita*

## Data Availability

Data sharing upon request to A.G.
